# Do Ionic Liquids Slow Down in Stages?

**DOI:** 10.1021/jacs.3c08639

**Published:** 2023-11-14

**Authors:** Bichitra Borah, Gobin Raj Acharya, Diana Grajeda, Matthew S. Emerson, Matthew A. Harris, AM Milinda Abeykoon, Joshua Sangoro, Gary A. Baker, Andrew J. Nieuwkoop, Claudio J. Margulis

**Affiliations:** † Department of Chemistry, The University of Iowa, Iowa City, Iowa 52242, United States; ‡ Department of Chemistry and Chemical Biology, Rutgers University, Piscataway, New Jersey 08854, United States; ¶ Department of Chemical and Biomolecular Engineering, University of Tennessee, Knoxville, Tennessee 37996, United States; § National Synchrotron Light Source II, Brookhaven National Laboratory, Upton, New York 11973, United States; ∥ Department of Chemistry, University of Missouri, Columbia, Missouri 65211, United States; # Department of Chemical and Biomolecular Engineering, The Ohio State University, Columbus, Ohio 43210, United States

## Abstract

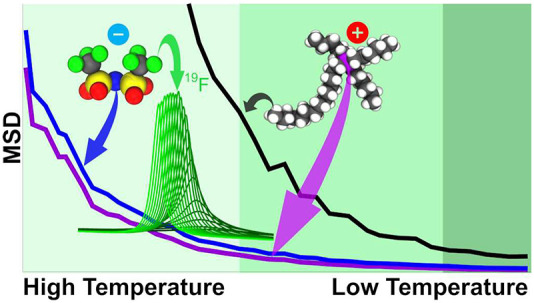

High impact recent articles have reported on the existence of a liquid–liquid (L–L) phase transition as a function of both pressure and temperature in ionic liquids (ILs) containing the popular trihexyltetradecylphosphonium cation (P_666,14_^+^), sometimes referred to as the “universal liquifier”. The work presented here reports on the structural-dynamic pathway from liquid to glass of the most well-studied IL comprising the P_666,14_^+^ cation. We present experimental and computational evidence that, on cooling, the path from the room-temperature liquid to the glass state is one of separate structural-dynamic changes. The first stage involves the slowdown of the charge network, while the apolar subcomponent is fully mobile. A second, separate stage entails the slowdown of the apolar domain. Whereas it is possible that these processes may be related to the liquid–liquid and glass transitions, more research is needed to establish this conclusively.

Recent studies^1,2^ have shown puzzling behavior close to the glass transition temperature (*T*_g_) for some ionic liquids comprising the P_666,14_^+^ cation. We use computer simulations in combination with solid-state NMR measurements to describe in atomistic detail the slowdown mechanism for P_666,14_^+^ coupled with the bis(trifluoromethylsulfonyl)imide (NTf_2_^-^) anion for which a L–L phase transition has been reported^1^ proximal in temperature to *T*_g_; we also use X-ray scattering to follow concomitant structural changes and dielectric spectroscopy measurements^3^ to better understand the behavior of the charge network. Specifically, we use Replica Exchange Molecular Dynamics simulations (REMD)^4,5^ combined with NMR measurements of strategically located atomic centers (^13^C, ^19^F, ^1^H) to identify the temperature regime in which subionic IL components associated with the charge network and the apolar domains go from a state of mobility to one of rigidity on our observation time scale. Computational and experimental details are provided in Sections S1 and S2 of the Supporting Information. We measure T_1_, T_2_, 1D line widths, and INEPT transfer efficiencies, all of which are sensitive to motion on the ps–ns time scale, ideal for detecting rotations around carbon–carbon bonds.^6^ These observables can establish the temperature at which specific local motions become slower than can be detected with the technique. We must be mindful that force fields for ILs do not necessarily match the experimental *T*_g_ ≈ 195 K.^1^ In our REMD study we observe a broad transition in the range ≈250–310 K as can be gleaned from plots of C_p_ vs *T* and ρ vs *T* in Figures S1 and S2, respectively; in other words, the lower temperature value in this range is 50–60 degrees above the experimental *T*_g_. Whereas we can observe a broad simulated transition region, even heroic REMD simulation efforts cannot unequivocally discern whether this is simply a glass transition or a combination of a glass transition and a L–L transition. The C_p_ in Figure S1 has significant noise, and in Figure S2, the density in the region 250–310 K can be fit in multiple ways. Hence, we do not attempt to distinguish within these plots multiple thermodynamic states beyond glass and liquid. Our NMR experiments were acquired between 240 and 325 K, a range which does not extend to the glass or L–L transition temperatures observed via calorimetry. Nonetheless, our experimental and computational data show that, as temperature decreases, the charge network and the apolar domain are each marching toward a slowdown transition sequentially as opposed to simultaneously.

Figure 1a shows 2D HC spectra acquired at 15 kHz MAS. Under these conditions at sample temperatures above ∼270 K, we observe liquidlike NMR behavior despite the high viscosity of this liquid. As we lower the temperature, we detect a stepwise loss of H–C signals, consistent with the T_2_ relaxation time falling below the ∼3 ms needed for this one bond H–C INEPT^7^ transfer (see Figure S3b,c for ^1^H T_2_ measurements). In these two-dimensional experiments we have assigned the chemical shifts of the carbons at each end of the alkyl tail using through-space ^1^H−^1^H mixing prior to the 2D HC readout^8^ (Figure S4). Thus, we are able to determine we lose signal first from ^13^C atoms adjacent to the charge network and only at lower temperatures for those atoms far from it. Below the temperature where motions have slowed to the point that INEPT is not effective, we can still monitor key atoms in the ions using direct excitation; see Figure 1b. As we lower the temperature, the signals from the charge network, ^19^F  on the anions and the ^13^C signal directly bonded to the P atom on the cation, broaden significantly (meaning they become more “solid-like”^9,10^) while the terminal ^13^C signal in the apolar domain remains narrow and in the liquid state. These changes in line width are consistent with the ^19^F T_2_ values (Figure S3a) and are mirrored by changes in the T_1_ relaxation times for which the minima report on motion.^11^ We observe the minimum of the T_1_ for ^19^F (Figure S5a) occurring at 260 K and the minimum for C6_6_/ C14 (Figure S5c) not yet reached at 240 K matching the trend observed for shorter chain ILs;^12^ at 240 K (about 45 degrees above *T*_g_), the terminal parts of alkyl tails are still in rapid motion. Reaching *T*_g_ would provide little extra information besides possibly seeing the last ^13^C peaks broaden; in addition, measuring T_1_ and T_2_ site specifically would become extremely challenging.

**Figure 1:**
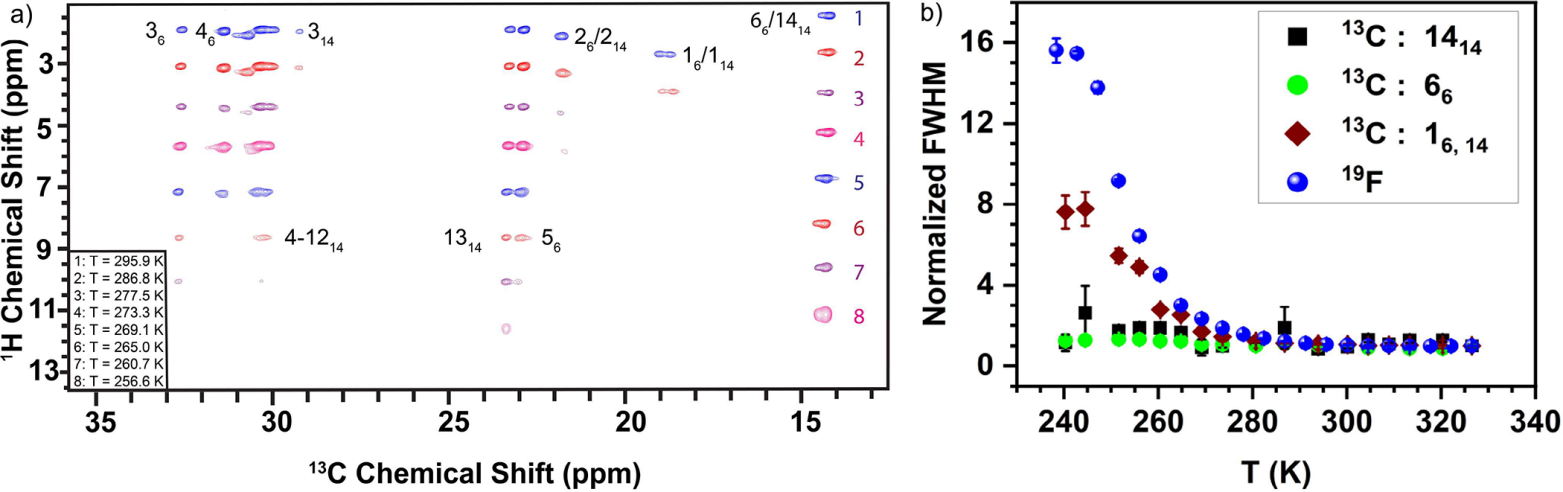
(a) 2D HC INEPT spectra at different sample temperatures offset to clarify peak differences. Carbons are labeled by chain number with the subscript indicating chain length. Assignments were done with ^1^H–^1^H through-space mixing experiments (Figure S4). Loss of signal is progressive down the chain as a result of decreasing T_2_ relaxation times, indicative of restriction in motion. The terminal methyl groups (6_6_/14_14_) are visible in all spectra, with the next carbon (5_6_/13_14_) starting to vanish at 260.7 K and barely visible at 256.6 K for 6 and 14 carbon chains, respectively. Panel b shows the full width half-maximum (FWHM) of 1D NMR peaks of ^19^F (anion) and ^13^C (tail components) (see Figure S6). FWHM changes with temperature due to shorter T_2_ and increased structural heterogeneity. The broadening of the ^19^F peak at a threshold temperature ∼260 K whereas the apolar tail stays narrow is therefore further evidence of the slowing of the charge network before the tails.

Figure 2, top and bottom, shows the structure function S(q) at selected temperatures produced from experiment and REMD simulations, respectively (see also Figures S7 and S8). These temperatures were selected to highlight thermodynamic states below, close to, and above the experimental and computational glass transition regions. In REMD simulations, cold and hot snapshots are swapped in order to facilitate proper thermodynamic sampling, particularly at low temperature. However, the technique may have its pitfalls when studying a glass^13^ as it may be “ergodizing” something that is not actually ergodic. REMD produces smooth S(q) changes as a function of temperature as opposed to regular MD starting from well equilibrated initial conditions where S(q) features, particularly those in the region 0.5–0.6 Å^–1^, appear to be very sensitive to the actual glass or low temperature liquid being trapped as can be gleaned from Figure S10 (*vide infra*). Figure 2 shows between 1 and 2 Å^–1^ what we have commonly termed an “adjacency” peak which is associated with all sorts of intramolecular and intermolecular interactions between atoms that are close by (this peak is present for all liquids, but ILs often have two other peaks at lower q value).^14−38^ Around 0.7–0.8 Å^–1^ there is what we have called a “charge alternation” peak linked with the spacing between positive moieties separated by an anion or with anions separated by positive charge.^14−38^ The lowest q-peak below ≈0.5 Å^–1^ is what is commonly described as a prepeak or a first sharp diffraction peak and is associated with the distance between charge networks spaced by apolar domains.^14−38^ Notice that at low temperature there is an additional small peak at around 0.5–0.6 Å^–1^ (blue bar). This peak has been previously observed experimentally in the glass regime,^22^ and we see it both in simulation and experiment. In examining the experimental data, we see that the peak becomes prominent close to the glass transition region and disappears upon further heating. Based on the set point temperatures of our measurements, this is a feature of the glass. However, we did not calibrate sample temperatures directly, as that would have required their destruction. Hence, we cannot exclude the 0.5–0.6 Å^–1^ peak which regular MD shows is very sensitive to the condensed phase configurations trapped (Figure S10), being also a feature of the incipient liquid forming at temperatures just above the glass transition (the so-called liquid-2).^1^ If we take well equilibrated configurations (see Section S2) and use them as initial conditions for constant pressure and temperature (NPT) MD runs of fixed duration (20 ns each), obtaining the average mean square displacement of selected atomic species at 10 ns, we find something quite intriguing. Below the simulated transition regime (defined with translucent green bars in Figure 3 and Figures S1 and S2), motion of all atoms whether part of the charge network or the tail domains is mostly arrested on this time scale, but in the low temperature portion of the transition region, the tail domains but not the charge network unlock and start moving. This is clearly seen from Figure 3 when we consider the black line corresponding to the P atom or the red line corresponding to anionic N in contrast to the green or blue lines corresponding to the C atoms away from the charge network in the apolar domain; this phenomenon is akin to other examples of dynamical heterogeneity for ILs that we have studied.^26,32,35,39−41^ As we consider C atoms that are closer to the charge network in the regime in which tails flail but the charge network is rigid, their MSD gets smaller and closer to that of the network (data not shown). Of course, the exact MSD will depend on the time duration considered, but the overall picture is clear. Both NMR experiments and simulations show that as we march from high to low temperature toward the experimental L–L and glass transition temperatures, there is a significant mobility gap between polar and apolar regions in this IL.

**Figure 2:**
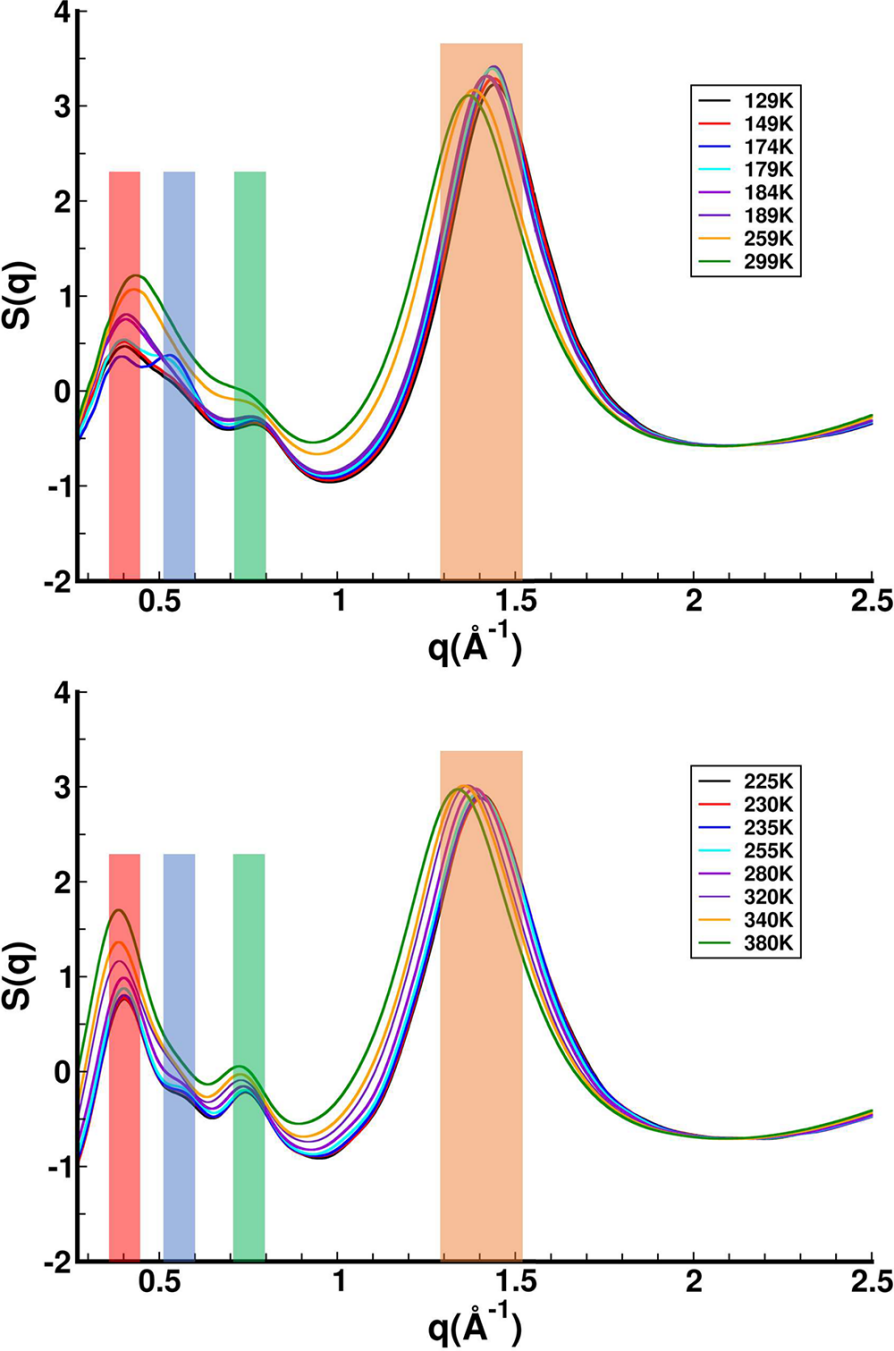
(Top) Selected S(q) functions in a temperature range that starts below *T*_g_ and finishes in the normal liquid region (Figures S7 and S8 provide S(q) in the full range of temperatures studied). (Bottom) Selected REMD S(q) functions in a temperature range that starts below the force field *T*_g_ and finishes in the normal liquid region. See Figure S9 testing the convergence of the REMD results. Bars in both subfigures: (red) prepeak, (blue) peak associated with glass and possibly the incipient liquid, (green) charge alternation peak, and (orange) adjacency peak.

**Figure 3:**
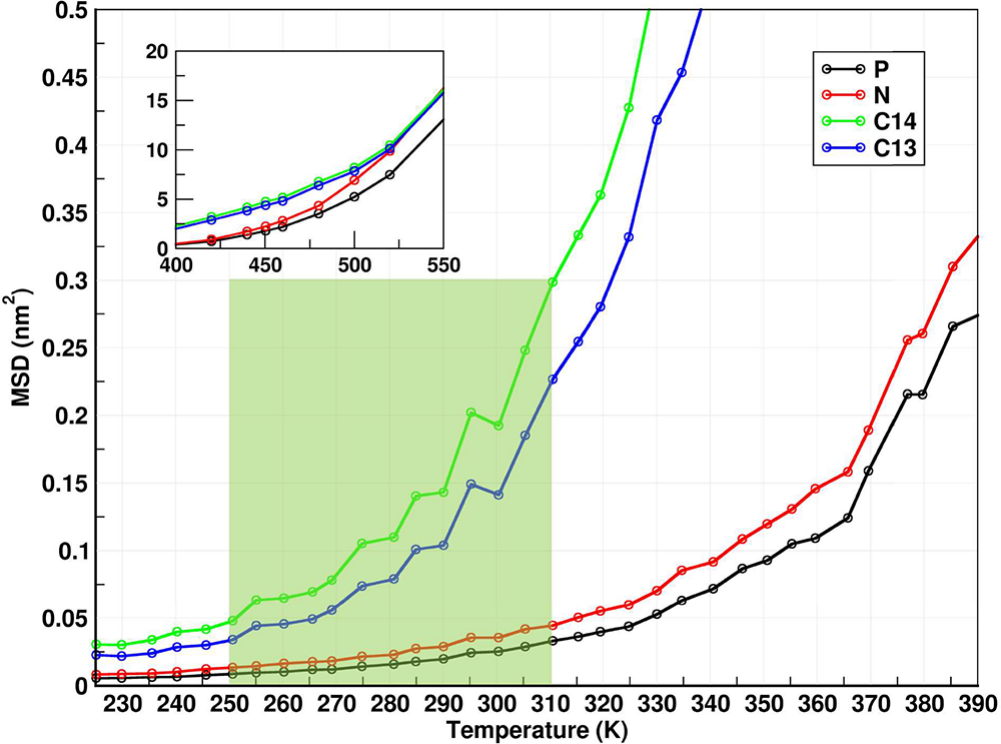
For the terminal cationic C in the longest tail (C14), C in the methylene adjacent to it (C13), N in the anion, and P in the cation from well equilibrated 20 ns NPT trajectories: The average mean square displacement at 10 ns. The inset shows the behavior of species at high temperature, and the translucent green bar is the transition region defined in Figures S1 and S2.

So what does this all mean? The way we interpret our results is by considering, as we go from low to high temperature, a first gradual process that involves unlocking the apolar portions of the liquid and a second, separate, gradual process at higher temperature that unlocks the charge network, making the IL more conductive. Stickel plots of the dc ionic conductivity (Figure S11)^3^ show that, consistent with a change in the dynamics of the charge network a few degrees above *T*_g_, there is a slope change in the derivative plot (see Section S4). Whether the unlocking of the apolar domains corresponds to the glass transition or the unlocking of the charge network relates in some way to the L–L phase transition cannot be fully resolved from Figure 3 or our NMR experiments; after all, we expect that structurally similar ILs will show these two processes even if they do not show a L–L transition. Yet, we also know (1) that, at the L–L phase transition, conductivity increases,^1^ and this is exactly what one would expect from the gradual unlocking of the charge network and (2) that, below *T*_g_, all motion—including from the apolar domains—should become more restricted, which again matches our observation.

Seen as a whole, MD and NMR experiments jointly indicate two distinct structural liquid regions, a charge network and an apolar domain, regions that slow down sequentially rather than simultaneously upon cooling, an outcome we find fascinating and broadly significant. We suspect that this finding is quite general for systems having significant apolar character.

## Supporting Information Available

Experimental Section; Computational Section; plots of simulated C_p_ vs temperature; simulated density vs temperature; T_2_ relaxation times; HC INEPT 2D spectra; T_1_ relaxation times; 1D NMR spectra of ^19^F, ^1^H, and ^13^C; experimental S(q) at different temperatures in their full range and in the range 0.25–2.5 Å^–1^; REMD convergence comparison of S(q) for different temperatures; S(q) from regular MD for different temperatures; Dielectric Spectroscopy Results section; and Stickel plots (PDF)
