# Supplementary material for: Do Ionic Liquids Slow Down in Stages?
Source: J Am Chem Soc. 2023 Nov 14;145(47):25518–22. doi: 10.1021/jacs.3c08639 (PMC10691361; doi:10.1021/jacs.3c08639)
Supplement: Supplementary file 1 — ja3c08639_si_001.pdf [file ja3c08639_si_001.pdf]

Supporting Information: Do Ionic Liquids Slow Down in Stages?

Bichitra Borah,[†,⊥] Gobin Raj Acharya,[‡,⊥] Diana Grajeda,[‡] Matthew S. Emerson,[†] Matthew A. Harris,[¶] AM Milinda Abeykoon,[§] Joshua Sangoro,[*,¶,#] Gary A. Baker,[*,‖] Andrew J. Nieuwkoop,[*,‡] and Claudio J. Margulis[*,†]

†*Department of Chemistry, The University of Iowa, Iowa City, IA 52242, United States*

‡*Department of Chemistry and Chemical Biology, Rutgers University, Piscataway, NJ 08854, United States*

¶*Department of Chemical and Biomolecular Engineering, University of Tennessee, Knoxville, TN 37996, United States*

§*National Synchrotron Light Source II, Brookhaven National Laboratory, Upton, NY 11973, United States*

‖*Department of Chemistry, University of Missouri, Columbia, MO 65211, United States*

⊥*Contributed equally to this work*

#*Department of Chemical and Biomolecular Engineering, The Ohio State University, Columbus, OH 43210, United States*

E-mail: sangoro.1@osu.edu; bakergar@missouri.edu; an567@chem.rutgers.edu; claudio-margulis@uiowa.edu

Contents

S1 Experimental Section **S-3**

S1.1 Sample Details and Broadband Dielectric Spectroscopy S-3

S1.2 NMR S-3

S1.3 Total X-ray Scattering Measurements S-5

S2 Simulations Section **S-6**

S3 Additional X-ray, Simulation, and NMR Figures **S-8**

S4 Dielectric Spectroscopy Results **S-17**

References **S-20**

S1 Experimental Section

S1.1 Sample Details and Broadband Dielectric Spectroscopy

Trihexyltetradecylphosphonium bis(trifluoromethylsulfonyl)imide with purity >98% was obtained from Iolitec GmbH. Samples were dried under vacuum (10^{-6} mbar) at 50°C for 24 hours prior to experiments. Broadband Dielectric Spectroscopy (BDS) measurements were performed using a Novocontrol High Resolution Alpha Dielectric Analyzer equipped with a Quatro Cryo-system for temperature control with an accuracy of ±0.1K. Samples were placed between two polished gold-plated brass electrodes (diameter, 20mm) in a parallel-plate configuration. The sample thickness was controlled by silica spacers with thicknesses of around 100 μm. Before starting the full measurement, the dielectric responses of samples were monitored under an inert N_2 atmosphere at 400K as a function of time until thermal equilibration was achieved. The dielectric measurements[1] were carried out in the frequency range from 1 mHz to 10 MHz during the cooling and heating cycles. The dc ionic conductivity (σ_0) was determined from the frequency-independent values of the real part of the complex conductivity function as described in previous literature.[2]

S1.2 NMR

Approximately 8 μL of ionic liquid was packed into a 1.6 mm NMR rotor using a Hamilton syringe inside a glove box (Ar atmosphere). ssNMR data were acquired on a Bruker AVANCE III HD 600 MHz spectrometer equipped with an HFXY 1.6 mm Phoenix NMR broad-band Magic Angle Spinning (MAS) probe tuned to HFPC mode. The tuning frequency of the probe for ^{1}H, ^{19}F, ^{31}P and ^{13}C nuclei were 599.540, 564.088, 242.698, and 150.759 MHz respectively with typical 90 pulse widths of 1.9, 2.8, 2.0 and 2.0 us respectively. The temperature was controlled using a Bruker BCU-X chiller calibrated externally to ethylene glycol with the same cooler and gas flow settings. The sample rotor was inserted to the probe and cooled with a gas flow of 2000 Lph using the chiller at strong power to reach the lowest possible temperature (235 K) of the system. All magic angle spinning (MAS) experiments were run at 15 KHz regulated by a Phoenix MAS controller. ^{1}H-1D,

spin-lattice relaxation (T_1) and spin-spin relaxation (T_2) spectra were acquired with 4 scans with recycle delay of 2.5 s. ^{19}F-1D, T_1 and T_2 spectra were acquired with 4 scans with recycle delay of 2.0 s. ^{13}C-1D spectra were acquired with 15000 scans for the lowest temperatures, 8192 scans at intermediate temperatures and 4096 at higher temperature with a recycle delay of 2.5 s. All ^{13}C-T_1 spectra were collected with 512 scans with recycle delay of 5.0 s. All the NMR data were acquired and processed at Bruker Topspin 3.6.5.

The linewidths reported from 1D spectra are from global fits of the spectra to the single Gaussian function with the exception of the terminal methyl - ^{13}C peak where we used two Gaussian functions to deconvolute the terminal methyl' carbon peak associated with hexyl (6) and tetradecyl (14) chain. The convergence limit of fit was 10^{-9} and the goodness of fit (R^2) was greater than 99%.

T_1 measurements were done via inversion recovery. Sixteen values of the delay (τ) were measured and amplitude/intensity $I(\tau)$ was used to determine T_1 relaxation time by fitting to the equation: $I(\tau) = I(0)[1 - 2 \cdot A \cdot \exp(\frac{-\tau}{T_1})]$. Here $I(0)$ is the value of the integrated intensity/amplitude at which $I(\tau)$ approaches at $\tau \to \infty$, and A is the fitting constant.[3] For ^{13}C- 7 values of τ in a range of 75 ms to 12 s were used. The uncertainty of the determination of T_1 for all nuclei was less than 5%.

T_2 was measured using a Hahn-Echo. Fourteen values of the delay (τ) were used and the amplitude/intensity $I(\tau)$ were fit to determine the T_2 relaxation with the equation: $I(\tau) = I(0)\exp(\frac{-\tau}{T_2})$ where, $I(0)$ is the value of the $\frac{1}{e}$ part of integrated amplitude/intensity at which $\tau \to 0$. The uncertainty of the T_2 determination for all nuclei was less than 5%.

^{13}C detected 2D HC spectra were acquired using a refocused INEPT pulse sequence with the delay set to 1/4 of a typical directly bonded H-C J-coupling (1.5ms). Indirect ^{1}H dimensions were typically aquired with a dwell of 133.3 us (2 rotor periods) at 256 rows for 17ms of evolution. At lower temperatures with shortened ^{1}H T_2s fewer rows were used. 90 kHz of SPINAL-64 decoupling were used on proton during acquisition. For experiments with mixing, between 10 and 500 ms of spin diffusion were used (not all data shown). No active ^{1}H recoupling was applied. ^{31}P decoupling during acquisition was used to confirm the assignment of the 1 carbon via the removal

of the one bond ^{31}P J-coupling.

S1.3 Total X-ray Scattering Measurements

We loaded approximately 0.2 mL of ionic liquid into an NMR tube of outer diameter of 2.99 $\pm$ 0.03 mm inside a Glove box under Ar atmosphere. The NMR tube was made with borosilicate glass, type 1 class A purchased from Sigma-Aldrich. The sample tube was sealed with grease inside the glove box and flame-sealed immediately after taking it out from the glove box before X-ray measurements. After flame sealing, the empty portion of the tube was cleaned using iso-propanol, air dried, and used for background measurement.

X-ray scattering measurements were carried out in capillary transmission geometry using a PerkinElmer amorphous silicon area detector with 200 $\times$ 200 μm pixels placed 456 mm downstream from the sample at the 28-ID- 1 (PDF) beamline of the National Synchrotron Light Source-II at Brookhaven National Laboratory. The setup utilized a 74.5 keV (λ = 0.1665 Å) X-ray beam. An Oxford Cryostream was used to control the sample temperature in the range (80 - 300 K). First the Cryostream was set and stabilized at 80 K, and then pointed towards the sample for fast cooling. The sample was kept at 80 K for 30 mins prior to starting data acquisition. For temperature stability, 2-minutes of equilibration was used at each temperature step before starting data acquisition.

Detector calibration was carried out using a Ni standard powder sample. Two dimensional diffraction data were radially integrated to obtain intensity vs q data using the pyFAI software package.[4] Here, q $= |\vec{k}_f - \vec{k}_i| = \frac{4\pi \sin\theta}{\lambda}$ represents the momentum transfer wave vector and 2θ represents the scattering angle. Data reduction was carried out to obtain the experimental X-ray structure function S(q) (Eq. S1) using the PDFgetX2 software package.[5]

$$\mathrm{S(q)} = \frac{I_{coh}(\mathrm{q}) - \sum_i x_i f_i^2(\mathrm{q})}{\left[\sum_i x_i f_i(\mathrm{q})\right]^2} \tag{S1}$$

In Eq. S1, i, x_i, and f_i represent the atomic species, atomic fraction, and corresponding X-ray

form factor, respectively.[6–10]

S2 Simulations Section

All molecular dynamics simulations were performed using the GROMACS-5.1.4 software package.[11–13] The simulation box contained 512 ion pairs and the Packmol software[14] was used to pack the initial configuration of the system. Force-field parameters for bonds, angles, dihedrals and Lennard Jones terms were adapted from the OPLS-AA and CL&P force fields.[15–20] The system was first energy-minimized and later equilibrated at 300 K by scaling charges at 1% for 20 ns (at 50 bar), 10% for 20 ns (at 50 bar), and finally 100% for 10 ns (at 1 bar) in the constant temperature and pressure (NPT) ensemble. The system was later subjected to simulated annealing (56 ns at 1 bar) in the same ensemble from 300 K to 700 K and down to 220 K at full charge. The V-rescale thermostat[21] (0.2 ps time constant) and Berendsen barostat[22] (1.0 ps time constant) were used during the equilibration steps. The Nosé-Hoover thermostat[23] (0.2 ps time constant) and Parrinello-Rahman barostat[24] (1.0 ps time constant) were used for the annealing run and all used the MD integrator as coded in GROMACS. The cutoff for all the non-bonded interactions was set to the default in GROMACS (which is 1 nm). Electrostatic interactions were computed using the Particle-mesh Ewald method[25,26] with 0.12 nm Fourier spacing and a fourth order interpolation.

Following the annealing step and using the same setup, Replica Exchange Molecular Dynamics (REMD) simulations (94 replicas) ranging in temperature from 225 K to 400 K using the temperature generator for REMD-simulations[27] were run. The initial configuration for REMD was taken from the annealing step at 300 K. The last 10 ns of a 20 ns run were used to compute the X-ray structure function S(q) defined as:

$$\mathrm{S(q)} = \frac{\rho_0 \sum_i \sum_j x_i x_j f_i(\mathrm{q}) f_j(\mathrm{q}) \int_0^{L/2} 4\pi r^2 \left(g_{ij}(r) - 1\right) \frac{\sin(\mathrm{q}r)}{\mathrm{q}r} dr}{\left[\sum_i x_i f_i(\mathrm{q})\right]^2}, \tag{S2}$$

where L is the simulation box length, $g_{ij}(r)$ is the pair distribution function for atoms of type i and j, ρ_0 is the number density, and $f_i(\mathrm{q})$, $f_j(\mathrm{q})$, x_i and x_j, have the same meaning as in Eq. S1. We

also extended our REMD simulations for an extra 20 ns to make convergence comparison graphs (see Figs. S1 and S9).

From 20 ns molecular dynamics runs in the NPT ensemble, we computed the mean square displacement as a function of temperature for specific atomic centers

$$\mathrm{MSD_i}(\mathrm{t}-\mathrm{t}') = \left\langle |\mathbf{r}_\mathrm{i}(\mathrm{t}) - \mathbf{r}_\mathrm{i}(\mathrm{t}')|^2 \right\rangle . \tag{S3}$$

In Eq. S3, $\mathbf{r}_\mathrm{i}(\mathrm{t})$ and $\mathbf{r}_\mathrm{i}(\mathrm{t}')$ are the ith particle positions at time t and time t' respectively, and $\mathrm{t}-\mathrm{t}' =$ 10 ns. For temperatures where we had REMD results (below 400 K), the initial configuration for the runs were taken from the respective last snapshot of 40 ns REMD runs. For temperatures above 400 K (which do not need REMD for proper sampling) the initial frame was taken from the annealing run.

S3 Additional X-ray, Simulation, and NMR Figures

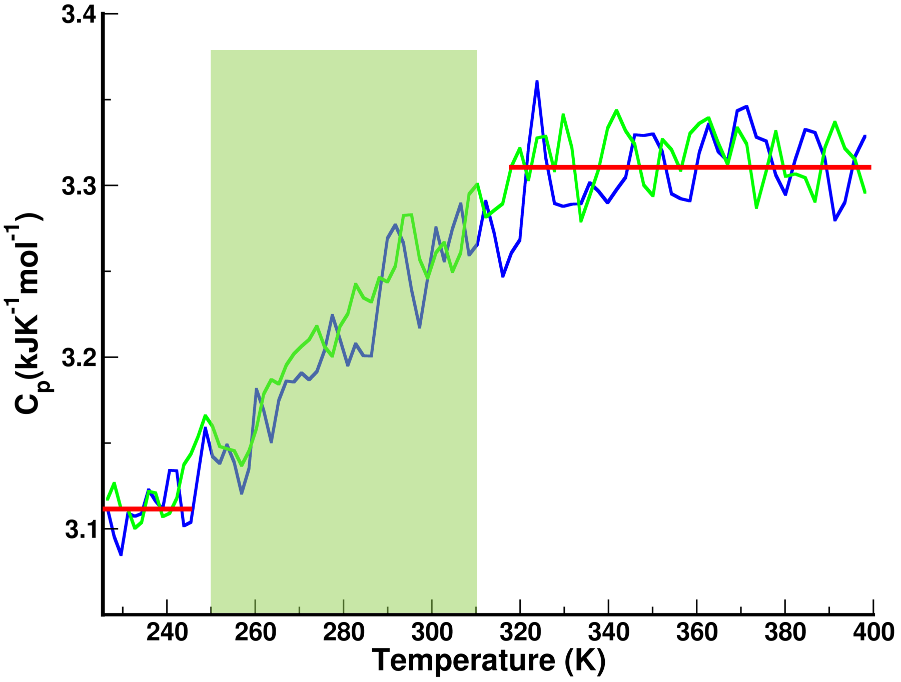

Figure S1: C_p vs. temperature from REMD simulations. The blue line is from last 10 ns of our production REMD trajectories and the green line is from our extension of these by 20 ns; the red horizontal lines are to guide the eye and the translucent green bar highlights what we consider to be the transition between glass and liquid. C_p was approximated as the center difference of average enthalpies for each replica produced by REMD using Xmgrace version 5.1.23. *Grace web page:* https://plasma-gate.weizmann.ac.il/Grace/ (accessed 6/29/2023).

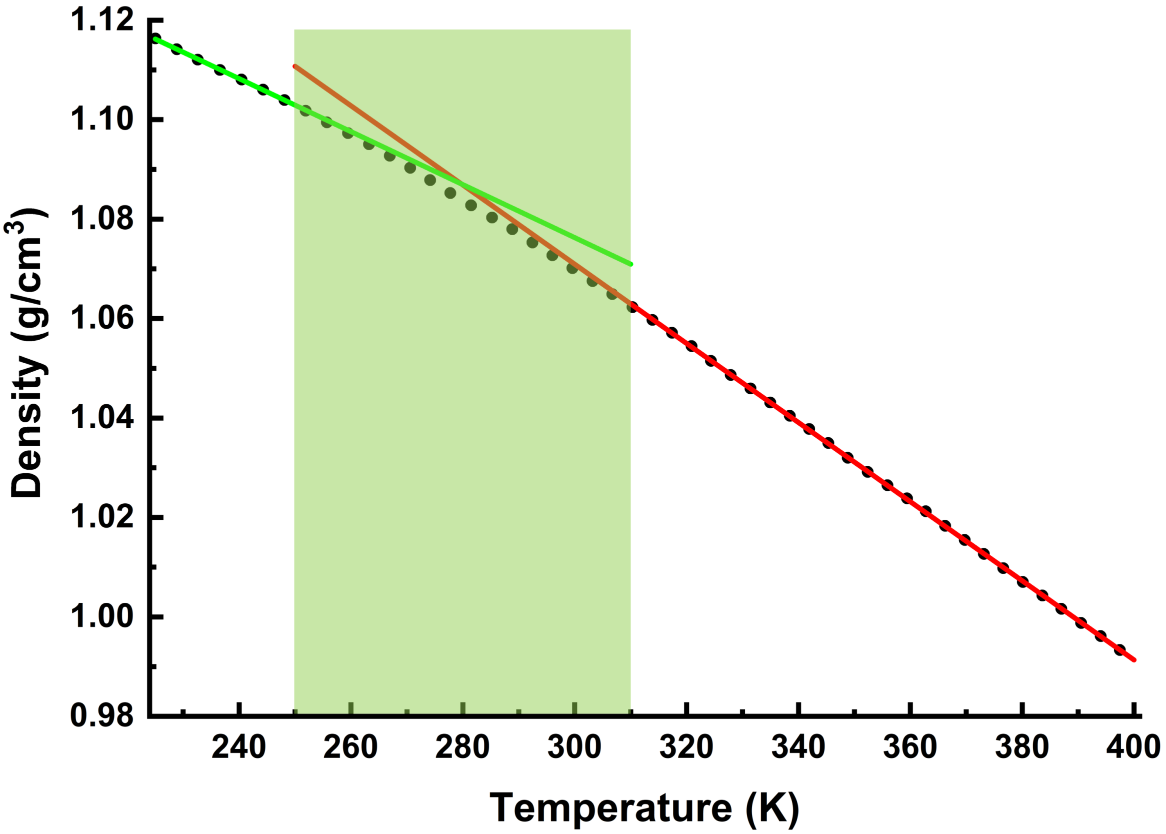

Figure S2: Average density vs. temperature computed from our REMD simulations. Green and red lines are fits to the low (225-245 K) and high (315-400 K) temperature regimes. The translucent green bar highlights what we are considering as the transition region between the glass and liquid.

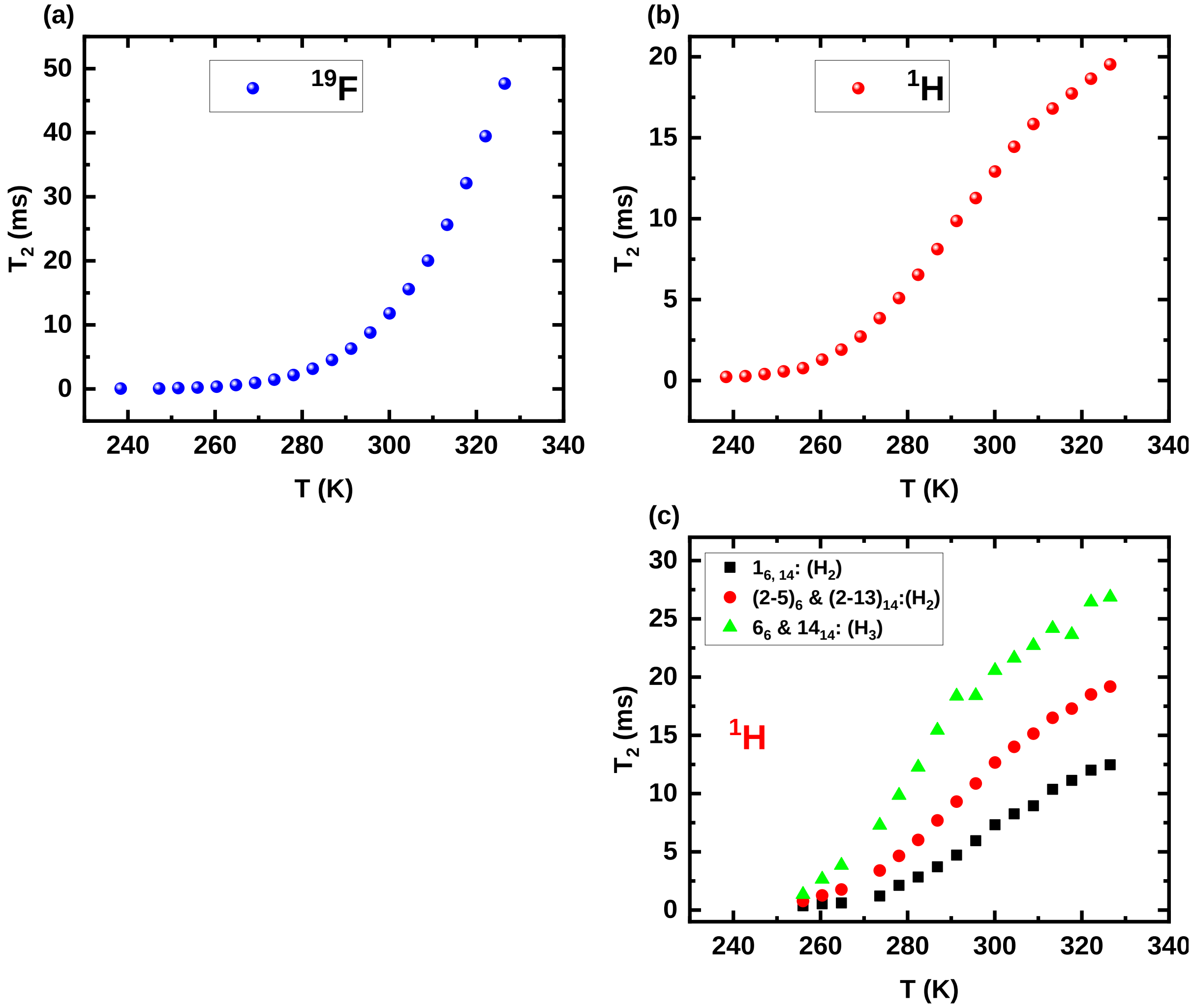

Figure S3: Bulk T_2 relaxation times of (a) ^{19}F, and (b) ^{1}H nuclei in $P^{+}_{666,14}/NTf^{-}_{2}$ measured as a function of calibrated sample temperature at 600 (^{1}H Larmor frequency) MHz NMR spectrometer at 15 kHz MAS. (c) ^{1}H-T_2 NMR relaxation times of selected hydrogen sites measured as a function of temperature. Analysis of the proton data was done by deconvolution of the 3 main areas of signal. For ^{1}H, the presence of multiple species (S6,b) can be seen in the shape of the T_2 curves with the clean decay of the ^{19}F curve contrasting with the multiple component decay seen for the ^{1}H. In the analysis of the proton data in part (c) the broadness of the lines (as seen in Fig. S6,b) mean that at colder temperatures deconvolution becomes challenging.

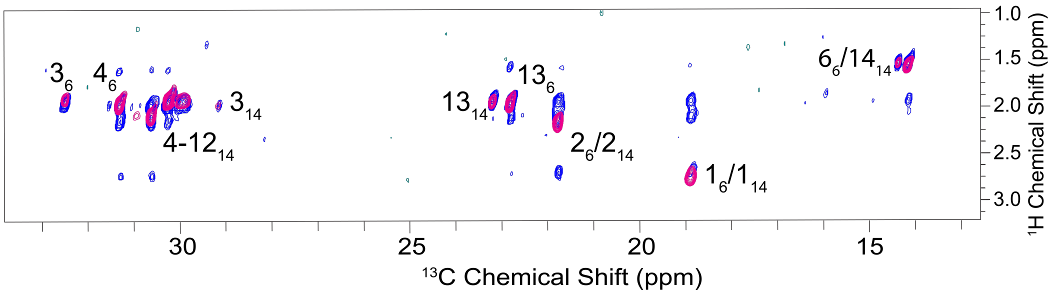

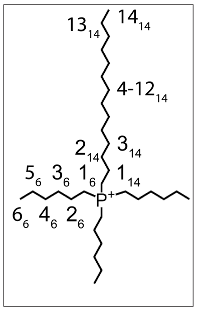

Figure S4: HC INEPT based 2D spectra with ^{1}H-^{1}H mixing to enable assignments. 80 ms of passive spin diffusion mixing (blue) results in cross peaks between the three closest carbon sites, relative to the HC 2D with no mixing (pink). The labeling scheme for the carbons on the cation are shown on the right for reference. ^{31}P decoupling resolved the doublet at 19 ppm seen in Fig. 1,a further confirming the assignment of the directly bonded carbon.

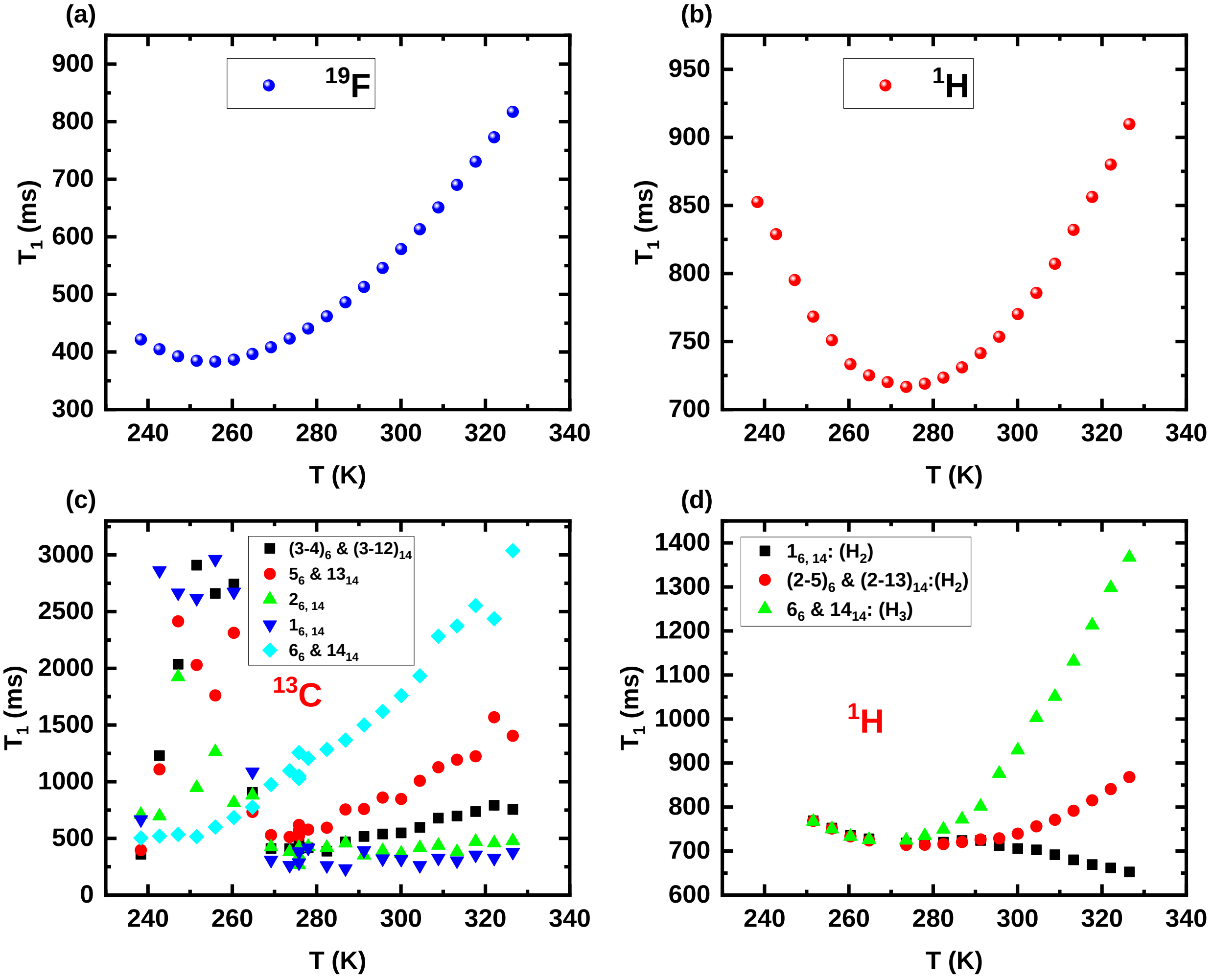

Figure S5: Bulk T_1 relaxation times of (a) ^{19}F, and (b) ^{1}H nuclei in $P^{+}_{666,14}/NTf_2^{-}$ measured as a function of calibrated sample temperature at 600 (^{1}H Larmor frequency) MHz NMR spectrometer at 15 kHz MAS. The minimum T_1 value for ^{19}F was reached around 255 K and indicates that at that temperature the motion of the CF_3 groups on the NTf_2^{-} ion have stopped on our observation time scale. For ^{1}H the minimum value at 273 K is the result of the superposition of the curves for multiple different sites as seen in S6,b and so the interpretation is less clear. (c) ^{13}C-T_1 and (d) ^{1}H-T_1 NMR relaxation times of selected carbon and hydrogen sites measured as a function of temperature respectively. While the analysis for most carbons is complicated in this series by the appearance of cold crystals, it is clear that the terminal methyl carbon (hexyl or tetradecyl tail) is more mobile than carbons closer to the P. Similar analysis of the proton data by deconvolution of the 3 main areas of signal show again that the methyl protons slow later than the rest, however, the broadness of the lines (as seen in Fig. S6,b) mean that at colder temperatures deconvolution becomes challenging.

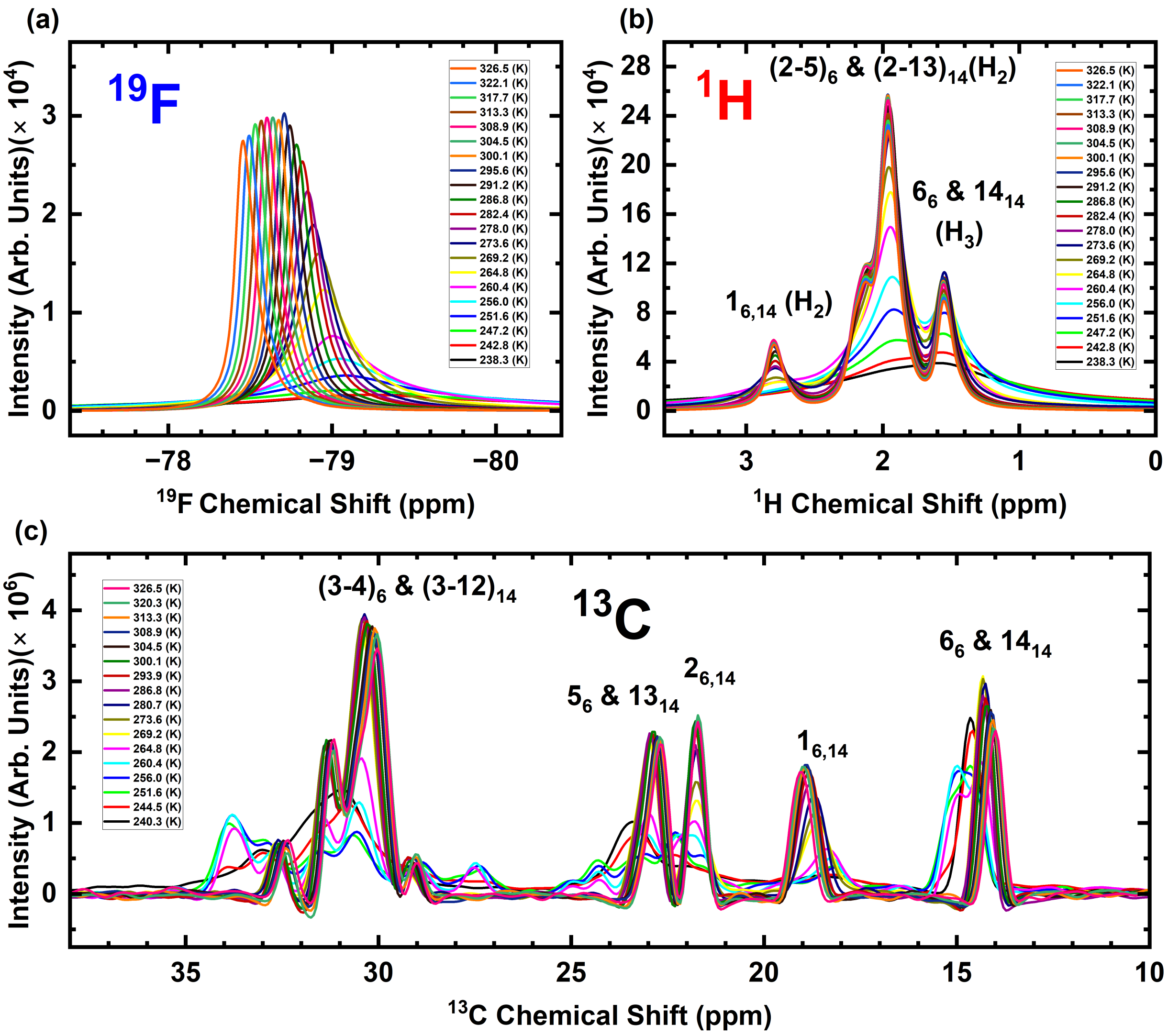

Figure S6: 1-D NMR spectra of (a) ^{19}F, (b) ^{1}H, and (c) ^{13}C nuclei measured on a 600 MHz (^{1}H Larmor frequency) NMR spectrometer at 15 kHz MAS. The full width half max (FWHM) lineshape parameter extracted from the peaks in these spectra are presented in Fig. 1. Between 245 K and 270 K a second set of peaks consistent with cold crystallization can been seen in the ^{13}C spectra. Overlap with these peaks meant that FWHM measurements were most accurate for the 1, 6_6, and 14_{14} carbons.

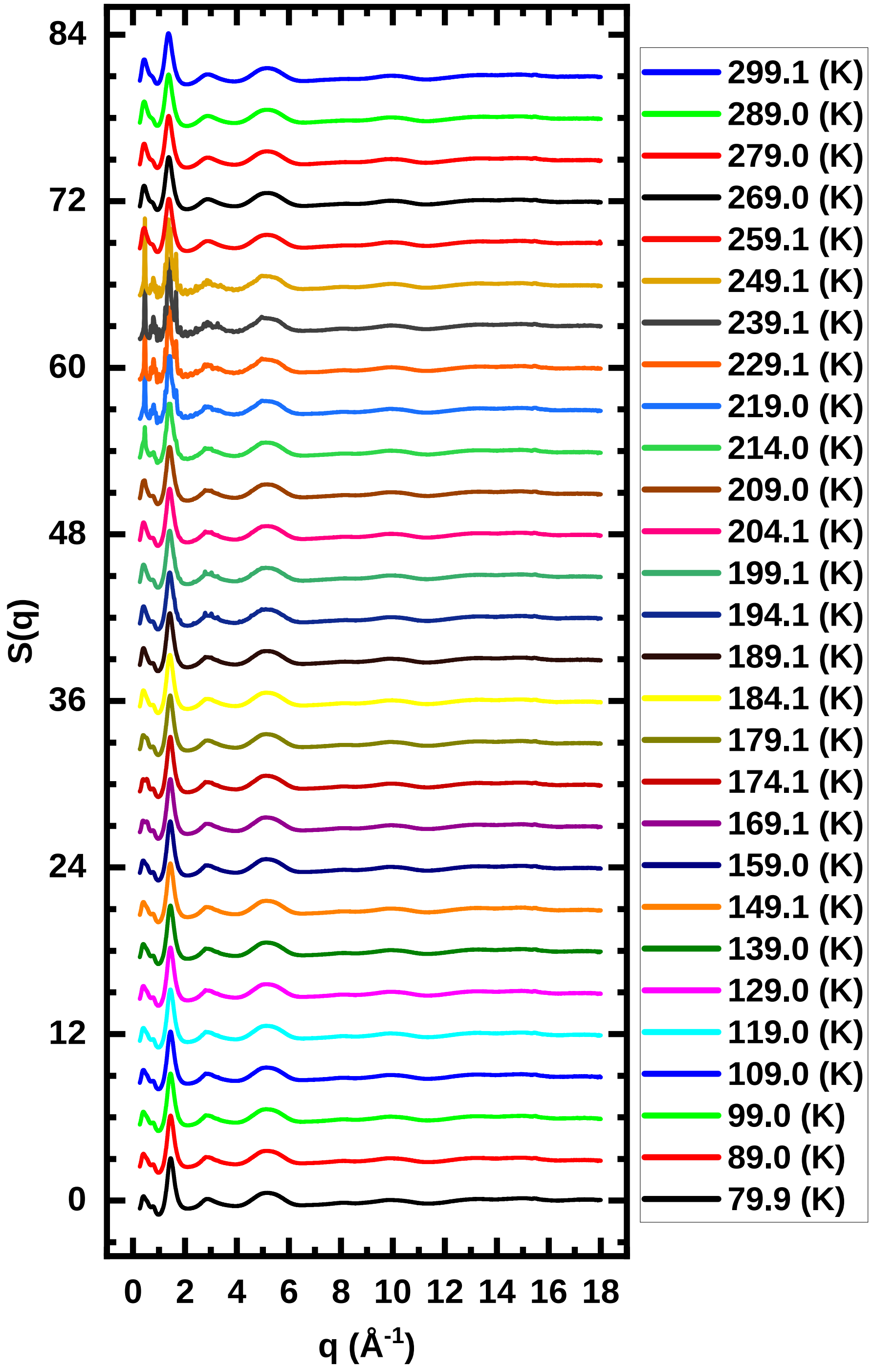

Figure S7: Experimental S(q) of $P^{+}_{666,14}/NTf_2^-$ at different temperatures. For clarity, a vertical offset of 3 is added to each function above 79.9 K.

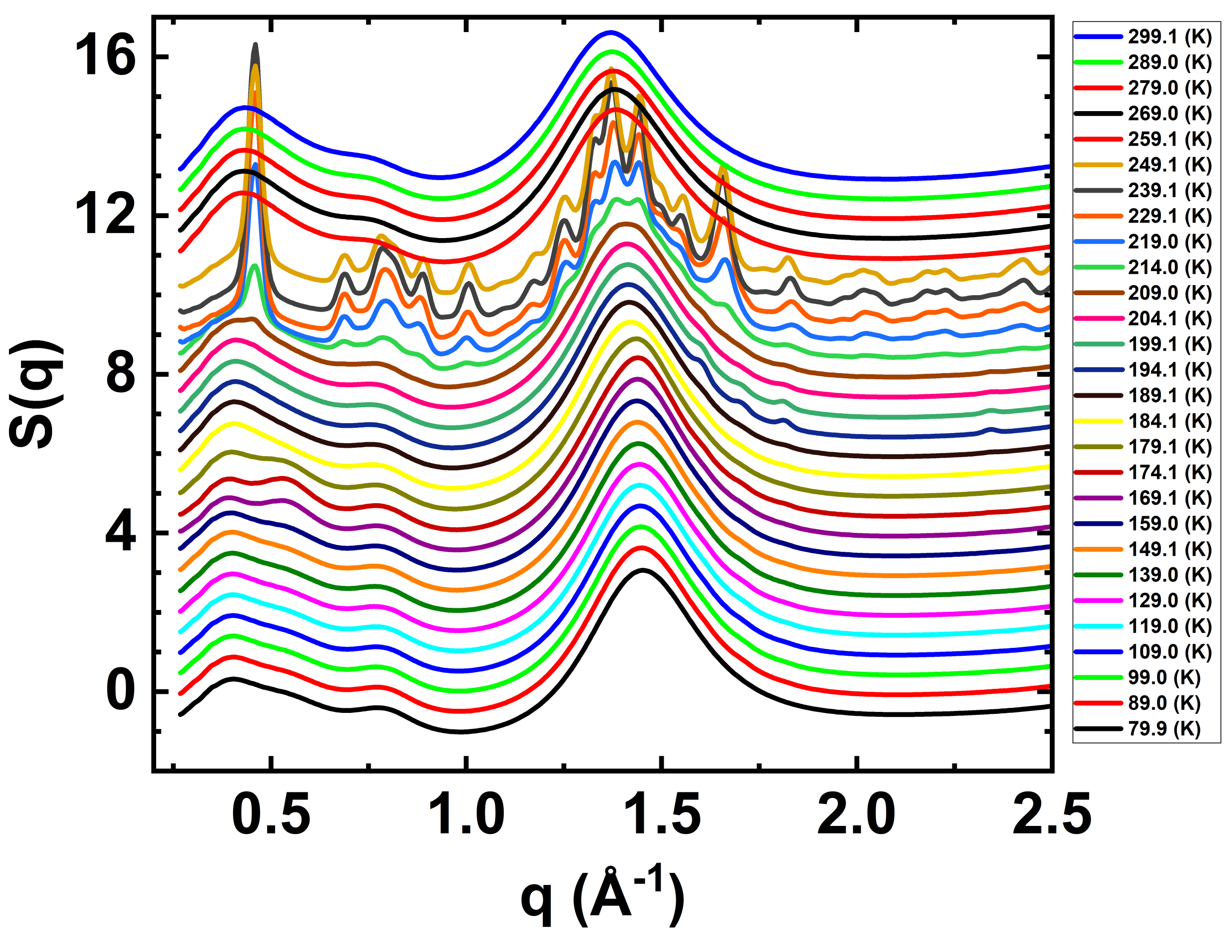

Figure S8: Same as Fig. S7 but in the relevant intermolecular q-range 0.25 to 2.5 Å^{-1}. In this case, the vertical offset between temperatures is set to 0.5. Notice that as we go from low to high temperature (glass to liquid), a feature at $\sim$ 0.5-0.6 Å^{-1} is present in the glass regime that becomes prominent and then disappears upon heating. In Fig. S10, we see from regular molecular dynamics simulations that this feature is very sensitive to the glass or low temperature liquid being trapped. REMD simulations "ergodize" the sampling and the feature shows up in the glass state and smoothly disappears upon heating (see Figures 2 (bottom) and S9). Notice also a regime in which cold crystallization is observed in the range $\sim$ 214-249 K.

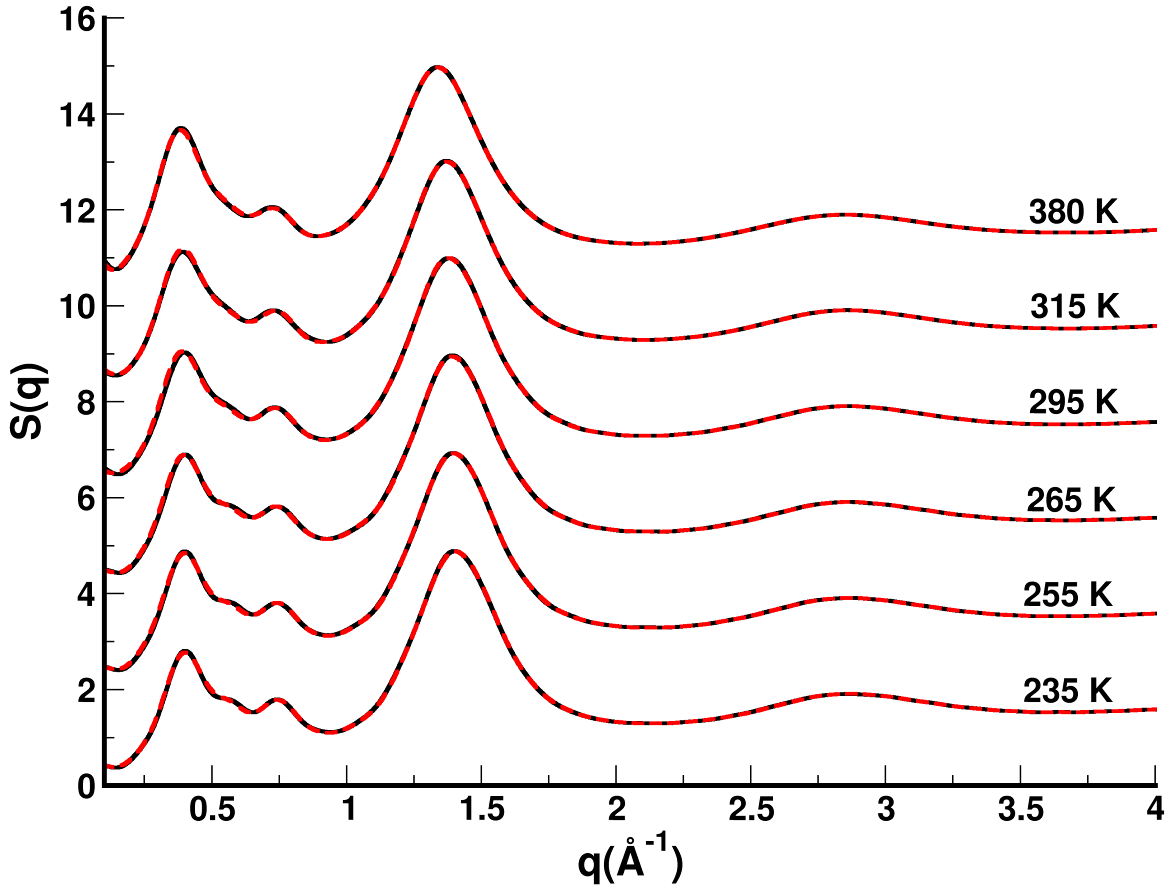

Figure S9: Comparison of S(q) computed from the production REMD run (black) and the extension of this run by 20 ns (red dashes) showing that results are almost identical except for very minor changes in the $\sim$ 0.5-0.6 Å^{-1} region. For clarity, each temperature has vertical offset of 2.

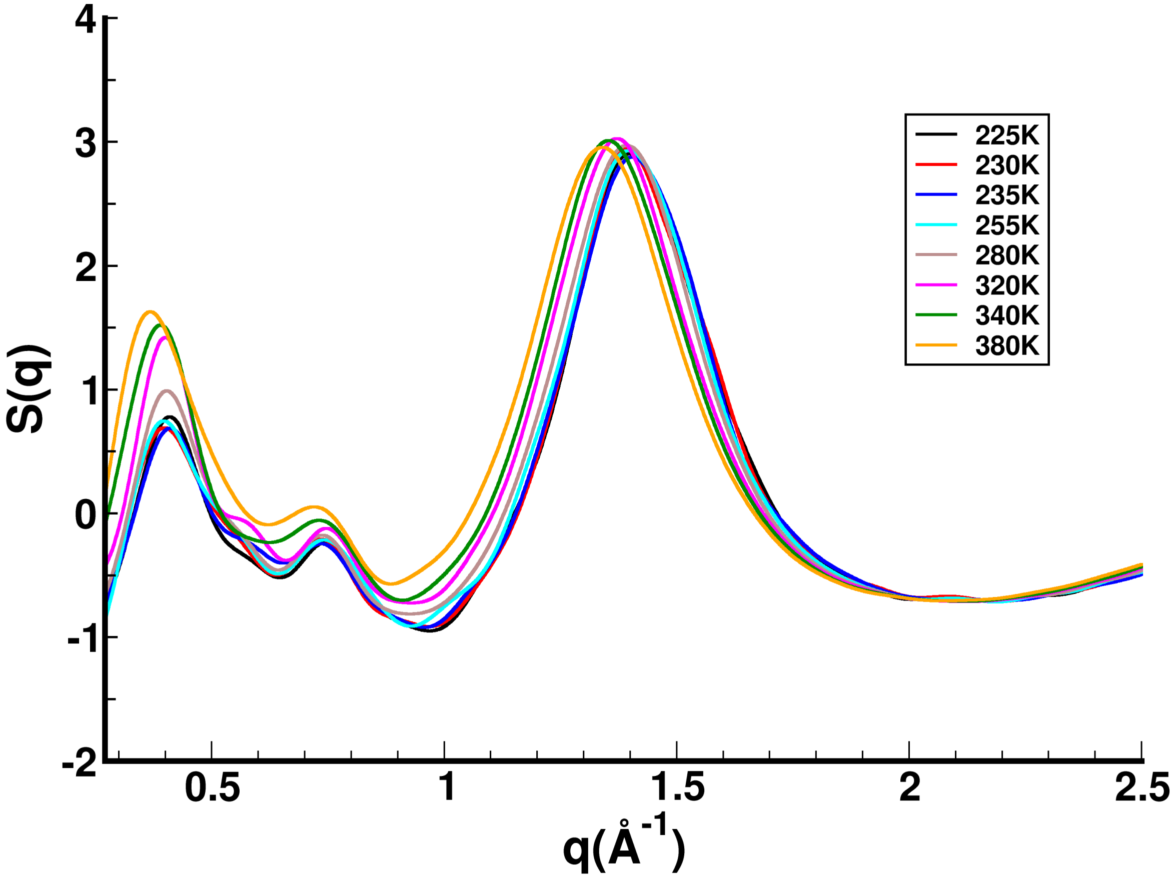

Figure S10: S(q) using regular NPT MD simulations (20 ns) after thorough equilibration using REMD. The figure shows that even well equilibrated inital conditions do not produce structure functions that have a consistent trend with temperature, particularly in the regime ∼0.5-0.6 Å^{-1}.

S4 Dielectric Spectroscopy Results

Many characteristic dynamic properties such as mean structural relaxation rates, diffusion coefficients, dc ionic conductivities, and zero-shear viscosity, exhibit a Vogel-Fulcher-Tammann (VFT) type of temperature dependence indicative of the increasing role of cooperative dynamics at lower temperatures. A change in the microscopic mechanisms driving dynamics in liquids can be accompanied by variation in the thermal activation. The Stickel derivative function (Eq. S4) provides an

intuitive means of illustrating these changes.

$$\Phi(T) = \left[-\frac{d(\log_{10}\sigma_0)}{d(1/T)} \right]^{-1/2} = (DT_0 \log_{10} e)^{-1/2} \left(1 - \frac{T_0}{T}\right) \tag{S4}$$

This is because the function has a linear dependence in inverse temperature with a negative slope for dynamics following VFT-like behavior. This differential analysis is commonly used as a model-free method of determining changes in dynamic behavior with regard to temperature. Here, D and T_0 are VFT parameters related to the fragility and ideal glass transition, respectively. In addition, $\log_{10} e$ denotes logarithm with base 10 of the exponential e. This representation is a useful method to accurately determine the VFT parameters, D and T_0, in cases where the dynamics can be described by the VFT equation. Figure S11 (bottom) shows $\Phi(T)$ as a function of inverse temperature, computed from σ_0 for $P^{+}_{666,14}/NTf^{-}_{2}$. It is immediately clear from the plot that the temperature dependence of σ_0 is well-described by the VFT equation based on the inverse linear dependence of the differential quantity with respect to inverse temperature. Furthermore, a dynamic shift in this ionic liquid is evident from a kink in the differential quantity corresponding to a change in slope. We conclude that the full temperature range of the conductivity data requires two sets of VFT parameters to accurately describe the dynamic behavior in this system, suggesting a possible change in the underlying mechanism at a characteristic temperature above the calorimetric glass transition temperature. It is possible that this dynamic crossover may be related to the L-L transition and we plan to explore this further in the future.

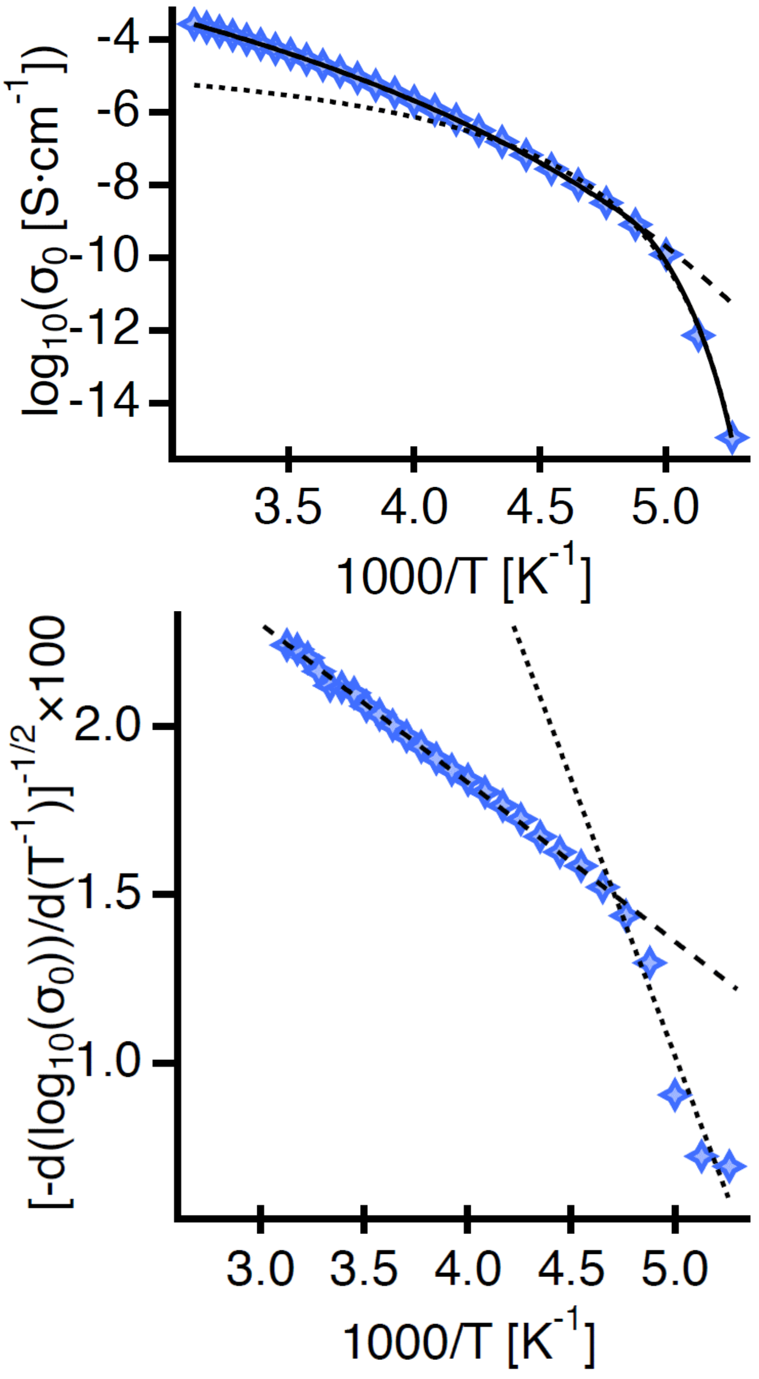

Figure S11: (Top) The log of the dc ionic conductivity as a function of inverse temperature and (bottom) Stickel derivative plot of the dc ionic conductivity for $P^{+}_{666,14}/NTf^{-}_{2}$. Dashed and dotted lines represent individual high and low-temperature fits using the Vogel-Fulcher-Tammann equation, respectively. Notice the change in slope in the derivative plot around the experimental L-L transition. Both figures adapted with permission from the thesis of Matthew Albert Harris.[1]

References

(1) Harris, M. A. Liquid-Liquid Transition in Ionic Liquids. Ph.D. Dissertation, University of Tennessee, 2022; `https://trace.tennessee.edu/utk_graddiss/7077` (accessed 9/28/2023).

(2) Harris, M. A.; Kinsey, T.; Wagle, D. V.; Baker, G. A.; Sangoro, J. Evidence of a liquid–liquid transition in a glass-forming ionic liquid. *Proc. Natl. Acad. Sci. U.S.A.* **2021**, *118*, e2020878118.

(3) Chung, S. H.; Lopato, R.; Greenbaum, S. G.; Shirota, H.; Castner, E. W.; Wishart, J. F. Nuclear magnetic resonance study of the dynamics of imidazolium ionic liquids with $-CH_2Si(CH_3)_3$ vs $-CH_2C(CH_3)_3$ substituents. *J. Phys. Chem. B* **2007**, *111*, 4885–4893.

(4) Kieffer, J.; Valls, V.; Blanc, N.; Hennig, C. New tools for calibrating diffraction setups. *J. Synchrotron Radiat.* **2020**, *27*, 558–566.

(5) Qiu, X.; Thompson, J. W.; Billinge, S. J. PDFgetX2: a GUI-driven program to obtain the pair distribution function from X-ray powder diffraction data. *J. Appl. Crystallogr.* **2004**, *37*, 678–678.

(6) Ogbodo, R.; Karunaratne, W. V.; Acharya, G. R.; Emerson, M. S.; Mughal, M.; Yuen, H. M.; Zmich, N.; Nembhard, S.; Wang, F.; Shirota, H.; Lall-Ramnarine, S. I.; Castner, E. W., Jr.; Wishart, J. F.; Nieuwkoop, A. J.; Margulis, C. J. Structural Origins of Viscosity in Imidazolium and Pyrrolidinium Ionic Liquids Coupled with the NTf2– Anion. *J. Phys. Chem. B* **2023**, *127*, 6342–6353, PMID: 37432303.

(7) Santos, C. S.; Annapureddy, H. V. R.; Murthy, N. S.; Kashyap, H. K.; Castner, E. W., Jr.; Margulis, C. J. Temperature-dependent structure of methyltributylammonium bis(trifluoromethylsulfonyl)amide: X ray scattering and simulations. *J. Chem. Phys.* **2011**, *134*, 064501.

(8) Kashyap, H. K.; Santos, C. S.; Annapureddy, H. V. R.; Murthy, N. S.; Margulis, C. J.; Castner, E. W., Jr. Temperature-dependent structure of ionic liquids: X-ray scattering and simulations. *Faraday Discuss.* **2012**, *154*, 133–143.

(9) Kashyap, H. K.; Hettige, J. J.; Annapureddy, H. V. R.; Margulis, C. J. SAXS Anti-Peaks Reveal the Length-Scales of Dual Positive–Negative and Polar–Apolar Ordering in Room-Temperature Ionic Liquids. *ChemComm* **2012**, *48*, 5103–5105.

(10) Dhungana, K. B.; Faria, L. F. O.; Wu, B.; Liang, M.; Ribeiro, M. C. C.; Margulis, C. J.; Castner, E. W., Jr. Structure of Cyano-Anion Ionic Liquids: X-ray Scattering and Simulations. *J. Chem. Phys.* **2016**, *145*, 024503.

(11) Hess, B.; Kutzner, C.; van der Spoel, D.; Lindahl, E. GROMACS 4: Algorithms for Highly Efficient, Load-Balanced, and Scalable Molecular Simulation. *J. Chem. Theory Comput.* **2008**, *4*, 435–447.

(12) Abraham, M. J.; Murtola, T.; Schulz, R.; Páll, S.; Smith, J. C.; Hess, B.; Lindahl, E. GROMACS: High performance molecular simulations through multi-level parallelism from laptops to supercomputers. *SoftwareX* **2015**, *1-2*, 19–25.

(13) Spoel, D. V. D.; Lindahl, E.; Hess, B.; Groenhof, G.; Mark, A. E.; Berendsen, H. J. C. GROMACS: Fast, flexible, and free. *J. Comput. Chem.* **2005**, *26*, 1701–1718.

(14) Martínez, L.; Andrade, R.; Birgin, E. G.; Martínez, J. M. PACKMOL: A package for building initial configurations for molecular dynamics simulations. *J. Comput. Chem.* **2009**, *30*, 2157–2164.

(15) Jorgensen, W. L.; Maxwell, D. S.; Tirado-Rives, J. Development and Testing of the OPLS All-Atom Force Field on Conformational Energetics and Properties of Organic Liquids. *J. Am. Chem. Soc.* **1996**, *118*, 11225–11236.

(16) Kaminski, G.; Jorgensen, W. L. Performance of the AMBER94, MMFF94, and OPLS-AA Force Fields for Modeling Organic Liquids. *J. Phys. Chem.* **1996**, *100*, 18010–18013.

(17) Canongia Lopes, J. N.; Pádua, A. A. H. Molecular Force Field for Ionic Liquids III: Imidazolium, Pyridinium, and Phosphonium Cations; Chloride, Bromide, and Dicyanamide Anions. *J. Phys. Chem. B* **2006**, *110*, 19586–19592.

(18) Canongia Lopes, J. N.; Pádua, A. A. H. Molecular Force Field for Ionic Liquids Composed of Triflate or Bistriflylimide Anions. *J. Phys. Chem. B* **2004**, *108*, 16893–16898.

(19) Gouveia, A. S. L.; Bernardes, C. E. S.; Tomé, L. C.; Lozinskaya, E. I.; Vygodskii, Y. S.; Shaplov, A. S.; Lopes, J. N. C.; Marrucho, I. M. Ionic liquids with anions based on fluorosulfonyl derivatives: from asymmetrical substitutions to a consistent force field model. *Phys. Chem. Chem. Phys.* **2017**, *19*, 29617–29624.

(20) Price, M. L. P.; Ostrovsky, D.; Jorgensen, W. L. Gas-phase and liquid-state properties of esters, nitriles, and nitro compounds with the OPLS-AA force field. *J. Comput. Chem.* **2001**, *22*, 1340–1352.

(21) Bussi, G.; Donadio, D.; Parrinello, M. Canonical sampling through velocity rescaling. *J. Chem. Phys.* **2007**, *126*, 014101.

(22) Berendsen, H. J. C.; Postma, J. P. M.; Van Gunsteren, W. F.; Dinola, A.; Haak, J. R. Molecular dynamics with coupling to an external bath. *J. Chem. Phys.* **1984**, *81*, 3684–3690.

(23) Nosé, S. A unified formulation of the constant temperature molecular dynamics methods. *J. Chem. Phys.* **1984**, *81*, 511–519.

(24) Parrinello, M.; Rahman, A. Polymorphic transitions in single crystals: A new molecular dynamics method. *J. Appl. Phys.* **1981**, *52*, 7182–7190.

(25) Darden, T.; York, D.; Pedersen, L. Particle mesh Ewald: An N·log(N) method for Ewald sums in large systems. *J. Chem. Phys.* **1993**, *98*, 10089–10092.

(26) Essmann, U.; Perera, L.; Berkowitz, M. L.; Darden, T.; Lee, H.; Pedersen, L. G. A smooth particle mesh Ewald method. *J. Chem. Phys.* **1995**, *103*, 8577–8593.

(27) Patriksson, A.; Van Der Spoel, D. A temperature predictor for parallel tempering simulations. *Phys. Chem. Chem. Phys.* **2008**, *10*, 2073.
